# Intraoperative neurophysiology in pediatric neurosurgery: a historical perspective

**DOI:** 10.1007/s00381-023-06155-0

**Published:** 2023-09-30

**Authors:** Francesco Sala

**Affiliations:** Section of Neurosurgery, Department of Neurosciences, Biomedicine and Movement Sciences, University Hospital, Verona, Italy

**Keywords:** Intraoperative neurophysiology, Neuromonitoring, Motor-evoked potentials, Cortico-cortical-evoked potentials, Pediatric neurosurgery

## Abstract

**Introduction:**

Intraoperative neurophysiology (ION) has been established over the past three decades as a valuable discipline to improve the safety of neurosurgical procedures with the main goal of reducing neurological morbidity. Neurosurgeons have substantially contributed to the development of this field not only by implementing the use and refinement of ION in the operating room but also by introducing novel techniques for both mapping and monitoring of neural pathways.

**Methods:**

This review provides a personal perspective on the evolution of ION in a variety of pediatric neurosurgical procedures: from brain tumor to brainstem surgery, from spinal cord tumor to tethered cord surgery.

**Results and discussion:**

The contribution of pediatric neurosurgeons is highlighted showing how our discipline has played a crucial role in promoting ION at the turn of the century. Finally, a view on novel ION techniques and their potential implications for pediatric neurosurgery will provide insights into the future of ION, further supporting the view of a functional, rather than merely anatomical, approach to pediatric neurosurgery.

## Introduction

Intraoperative neurophysiology (ION) has emerged over the past three decades as a discipline in clinical neurosciences aimed to detect and prevent an impending injury to the nervous system during neurosurgical procedures. Although ION was pioneered already in the 1970s with the advent of somatosensory evoked potentials (SSEPs) [1] and, a decade later, brainstem auditory evoked potential (BAEPs) [2], it was only in the mid-1990s that the field bloomed, particularly thanks to the advent of intraoperative motor evoked potentials (MEPs).

Most of these techniques are typically used in clinical neurophysiology to diagnose or monitor the evolution of a disease over time, for example, electroencephalography (EEG) to investigate epilepsy, electromyography (EMG) to study and monitor neuromuscular diseases, and somatosensory evoked potentials (SSEPs) to study spinal cord conductivity in demyelinating diseases such as multiple sclerosis.

In ION, these techniques are implemented and tailored for their intraoperative use. Here, the main limitations are the, electrically wise, very noisy environment where there is a high risk for artifacts interfering with physiological signals and, on the other hand, the fact that patients are under general anesthesia. This latter can remarkably impact the monitorability of evoked potentials and represent for many years a burden to the development of ION since anesthetic agents can profoundly affect neurophysiological monitoring.

The main goals of ION could be summarized as the following:

1. To detect an impending injury to the nervous system in time to be reverted or minimized by corrective measures, therefore avoiding severe, permanent, neurological deficit
2. To teach the surgeon about the detrimental effects of seemingly harmless surgical maneuvers
3. To reassure the surgeon on the safety of specific surgical maneuvers
4. To predict neurological outcome

Last but not least, in recent years, ION has also become a source of documentation for medico-legal issues.

Interestingly enough, pediatric neurosurgery has represented a very fertile field for the development of ION, with some of the most revolutionary techniques in ION being developed in the context of pediatric neurosurgery.

This article reviews the evolution of ION specifically in the field of pediatric neurosurgery, by highlighting some of the most relevant contributions in the brain, brainstem, spinal cord, and cauda equina surgery.

## Brain surgery (Fig. 1)

**Fig. 1:**
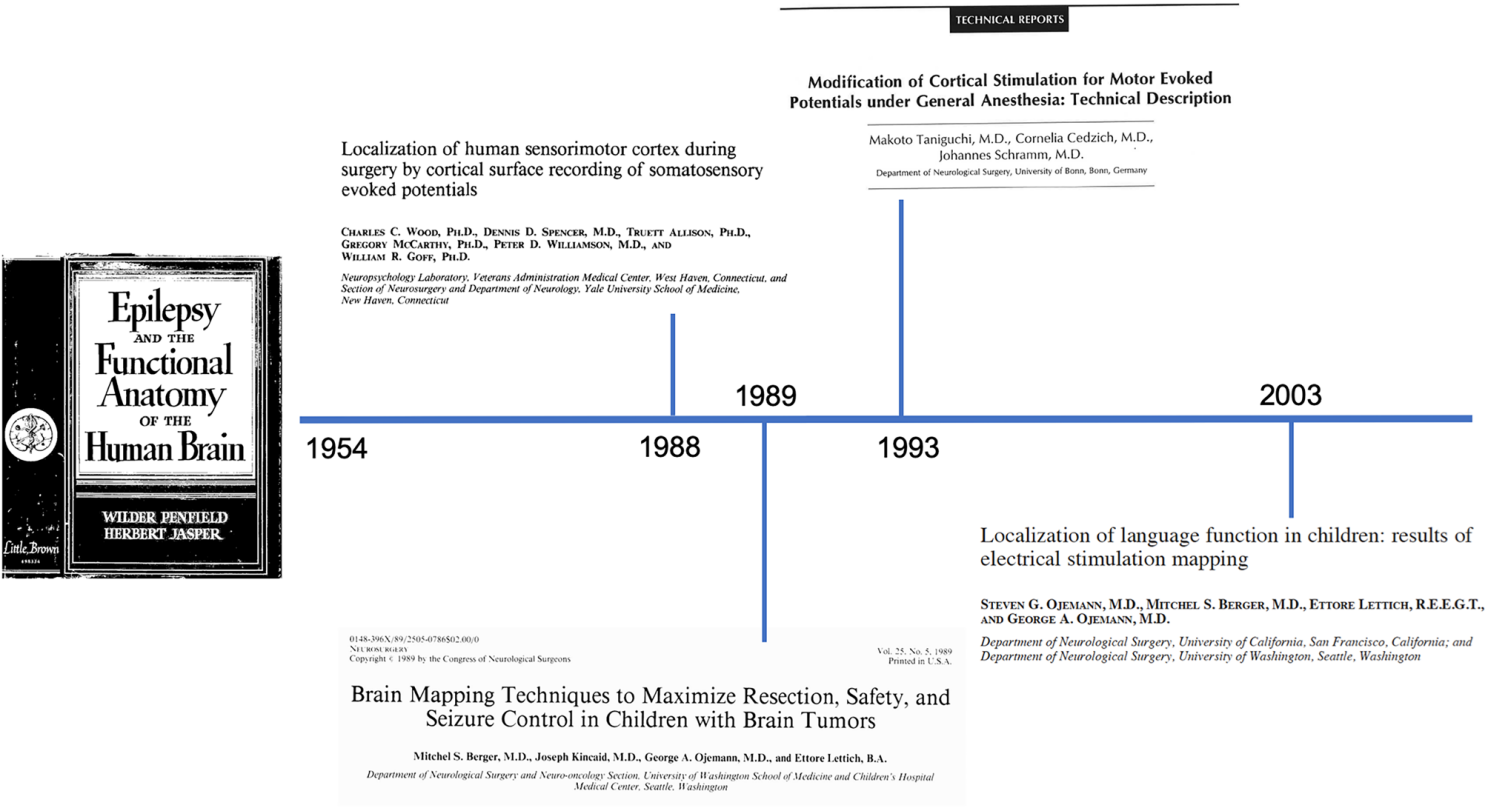
Timeline of ION development in brain tumor surgery

Although the first report on electrical stimulation of the human brain dates back to 1874 by Bartholow [3], Penfield was the one who pioneered the use of awake craniotomy and direct cortical stimulation (DCS) during epilepsy surgery to localize the epileptogenic zone by inducing auras, motor responses or seizures [4]. Almost 40 years later, in the 1970s, George Ojemann refined Penfield’s technique and introduced a more systemized language and neuropsychological testing to identify and spare functional brain areas [5, 6]. Progressively, the use of DCS shifted from epilepsy to brain tumor surgery, with an increasing interest also for subcortical stimulation. This latter has characterized the last decade due to the advent and widespread use of tractography, as well as the novel concept of the connectome and subcortical functional boundaries [7].

In pediatric neurosurgery, the first report of the use of DCS is also attributed to Penfield [8]. In a 4-year-old girl with tuberous sclerosis, he recorded by electrocorticography (ECoG) a well-localized spike focus over the right mid-central region of her primary motor cortex. By performing DCS of this area to reproduce her seizures and auras, Penfield elicited a sensation in the left hand, followed by a left clonic seizure. Following Penfield’s report, the use of DCS in children remained anecdotal for many decades, being used mainly in epilepsy surgery, but never systematically in brain tumor surgery.

Only in the late 1980s did Berger et al. address for the first time the value of cortical brain mapping to optimize the extent of resection and seizure control while reducing morbidity in children with brain tumors [9]. This was a seminal paper emphasizing the potential value of cortical stimulation in pediatric neurosurgery. However, as it was standard at that time, the classic technique for DCS was the use of a biphasic 50 or 60–Hz stimulation, sustained for several seconds and applied through the use of a bipolar probe. This is classically known as Penfield’s technique or, more recently, as “low frequency” stimulation, and it still represents the gold standard when it comes to language and cognitive mapping during awake craniotomies [10]. In the old days, it was used also to map the motor cortex but lately, a much more efficient technique was introduced.

In the mid-1990s, a German group in Bonn, led by Prof. Johannes Schramm, was on the frontline of ION, further developing or introducing brand-new neurophysiological techniques to localize the motor cortex under general anesthesia. One technique, the so-called phase-reversal technique, was introduced first by Wood et al. in 1988 and largely utilized by the Bonn’s group [11, 12]. By using contralateral median nerve stimulation and recording from a strip electrode placed perpendicularly across the supposed central sulcus, it was possible to localize the Rolandic fissure and, indirectly, the primary motor cortex [11].

The phase-reversal technique was particularly useful in pediatric neurosurgery due to the very low success rate of the classic Penfield technique for direct cortical stimulation.

The phase-reversal technique is used for central sulcus identification, based on the principle that the polarity of the SSEPs waveform is reversed when the recording electrodes are moved from the primary sensory cortex to the primary motor cortex, across the central sulcus. Due to the cytoarchitecture of the central sulcus, a mirror-image waveform with reversed potentials is typically recorded from the contacts overlying the primary motor cortex. Although modern pre-operative functional neuro-imaging including navigated transcranial magnetic stimulation and tractography may already provide reliable information to localize the primary motor cortex, still, the phase-reversal technique may be of particular value in younger children where DCS may be unsuccessful due to the immaturity of the descending motor pathways.

Before the advent of MEPs, the phase reversal technique was the only alternative to DCS with the Penfield technique to localize, although indirectly, the primary motor cortex. The Penfield technique had a very low success rate in children, particularly below the age of 5–6 years (see Table 1). This was never elucidated mainly because, even nowadays, we do not know much about the mechanism eliciting muscle contraction using Penfield’s stimulation parameters for DCS. Yet, it was a matter of fact that all major pediatric epilepsy surgery groups using this technique reported very low success rates.

**Table 1:** Summary of studies reporting on the effectiveness of Penfield’s, low frequency, mapping technique in children

Berger 1990 [13]	Electrically unexcitable cortex in children younger than 5–7 years
Nespeca 1990 [14]	No responses in children less than 3.8 years
Duchowny 1993 [15]	No responses in infants < 1 year 18% response in children aged 4–5 years 51% response in children aged 8–9 years
Chitoku 2001 [16]	Inverse relationship between amperage threshold and age using 50 Hz 0.2 ms duration bipolar technique
Signorelli 2004 [17]	Higher threshold in children younger than 5 years using 50 Hz 0.5 ms duration bipolar technique (mean 9.1 vs 3.7 mA)

We are indebted to Schramm’s group for the introduction, in the mid-1990s, of the so-called short train (or train-of-five) technique to elicit motor-evoked potentials under general anesthesia. The seminal paper was published in *Neurosurgery* in 1993 by Taniguchi et al. who showed that a short train of 3–5 electric pulses with an inter-pulse interval of 2–4 ms applied directly to the human motor cortex evokes a muscle MEP under general anesthesia [18].

While this technique became rapidly popular in Europe, it was not used in North America until 2002 when stimulators to perform multipulse transcranial electrical stimulation finally received FDA approval. Therefore in North America, most pediatric neurosurgery groups continued to use the traditional bipolar low frequency (50–60 Hz) Penfield’s technique until the end of the first decade of the new century.

In 2002, we published the first review paper on the state of the art of ION in pediatric neurosurgery [19], revealing that the vast majority of the literature referred to epilepsy rather than brain tumor surgery, and reports on cortical and subcortical mapping in children were anecdotal. We wrote: “While we are not aware of published data on MEP monitoring during brain surgery exclusively in pediatric series, our personal experience with extending the above-mentioned technique for use with children has been satisfactory. In particular, we have successfully performed neurophysiological monitoring and mapping in children less than 1 year of age by using the multipulse stimulation technique to elicit mMEPs.”

Interestingly, in a North American review paper on direct cortical stimulation in children published in 2009, still, only Penfield’s technique was mentioned [20]. Since Penfield’s technique allows mapping but not continuous monitoring of MEPs, the late introduction of Taniguchi’s technique in pediatric neurosurgery explains why, for many years, reports on cortical MEP monitoring in children remained exceptional. Therefore, despite MEP monitoring being performed largely during brain surgery in adult patients [21, 22], virtually no data existed in the pediatric age group.

In 2010, an abstract was presented at the 22nd ESPN Congress in Antalya by Korn et al. where, for the first time, DCS performed with both Penfield’s and Taniguchi’s techniques were compared in a group of 12 children with a main age of 4.7 years. The success rate was 92% for the short train technique versus only 17% using Penfield’s technique [23].

These preliminary data were corroborated by a much larger series in 2020. In 57 children who underwent supratentorial surgery, the success rate for motor mapping was 84% for the short train technique versus 25% for Penfield’s technique. Also, the youngest age at which motor mapping was successfully achieved was 93 versus 3 months, respectively, for Taniguchi’s and Penfield’s techniques [24].

Over time, the advantages of the train-of-five technique for DCS were also progressively acknowledged among pediatric neurosurgical centers in North America both in tumor surgery and epilepsy surgery [25, 26]. In 2018, a study from Sick Kids in Toronto compared conventional extra-operative motor mapping using Penfield’s technique with intra-operative cortical motor mapping using the train-of-five technique. Rather than extra-operative versus intra-operative motor mapping, what really matters in this study is the comparison of the two different cortical mapping techniques. Conclusions were that Penfield’s technique frequently led to after discharges and seizures and, with regard to motor mapping, was less informative than the train-of-five technique.

Therefore, when it comes to cortical and subcortical motor mapping, the past three decades have seen the progressive consolidation of the short train technique, first in Europe then in North America, and the simultaneous progressive sundown of Penfield’s technique.

Awake craniotomy is a standard procedure when mapping of cognitive functions is needed, in particular for language assessment. Yet, in children, awake craniotomy is of limited use for obvious reasons. In their seminal study on awake craniotomy, Pasquet and Penfield in 1954 concluded that children under 10 years were not suitable candidates [27]. This dogma remained undisputed until these days. In a recent review, it was observed that, unlike adults, awake surgery has yet to be accepted as the standard of care in pediatric patients, especially in the preadolescent groups [28]. Only 9 cases of children below the age of 11 years were reported in the last 65 years, 5 of those from a very recent series from Sick Kids in Toronto and, of the remaining four, two were procedures for deep brain stimulation [29]. But also among adolescents (11–19 years), who are presumed to be psychologically and intellectually more matured, only 49 cases of awake craniotomies have been reported in the literature [28].

Therefore, as a valuable alternative, the use of subcortical grids for extra-operative mapping has been extensively used. Ojemann, in 2003, reported 26 children, aged 4 to 16 years, where language mapping was performed either intra-operatively (DCS, 8 cases) or extra-operatively (grids, 18 cases) [30]. Apart from substantial variability in the localization of language sites, what emerged from that study was the paucity of language sites in the perisylvian cortices of children, as compared to previous studies in older patients. Similarly, within the pediatric age group, those younger than 9 years presented much less functional sites in the middle temporal gyrus and the perisylvian frontoparietal cortices, as compared to the older group which had a distribution more similar to adults. The authors concluded that with advancing age, maturational processes contribute to new foci of the cortex essential for language.

With time, particularly in the last decade, indications for awake craniotomy in children have enlarged, and studies focusing on the neuropsychological and psychiatric implications of this procedure in such a vulnerable patient population are rather encouraging with no evidence of significant stress disorders, likely supporting a more extensive application of awake surgery in children [31, 32].

## Brainstem surgery (Fig. 2)

**Fig. 2:**
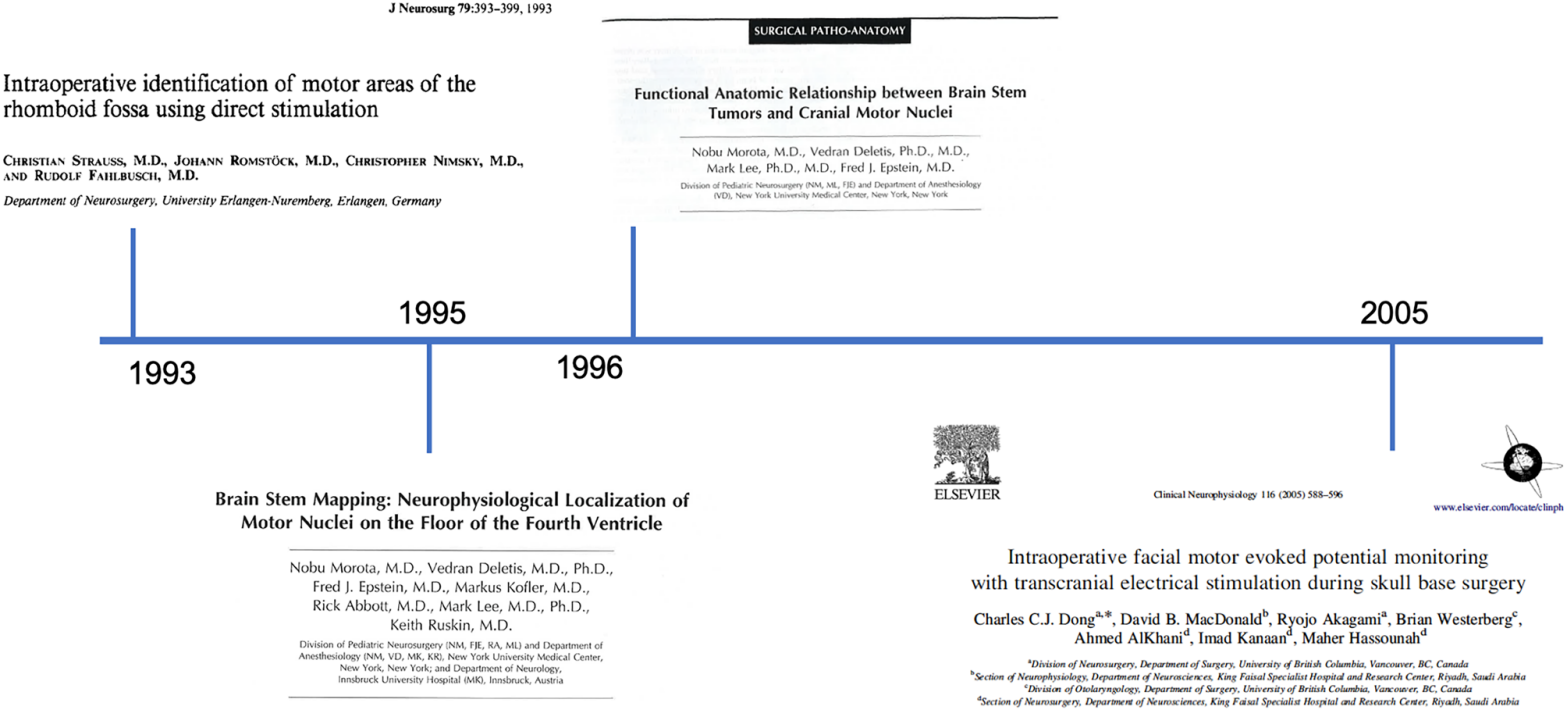
Timeline of ION development in brainstem surgery

Neurosurgeons have always paid a lot of respect to the brainstem, which, since the times of Cushing and Matson, was considered a minefield where no surgery was attempted for the high mortality and morbidity. As late as 1969, Matson wrote that “regardless of specific histology, brainstem gliomas must be classified as malignant tumors since their location in itself renders them inoperable” [33]. Indeed, the concentration of highly functional neural structures within the brainstem is such that even a small lesion can expose to severe neurological deficits, some of these being life-threatening [34].

Interestingly enough, it was mainly thanks to pediatric neurosurgeons such as Harold Hoffman in Toronto, Fred Epstein in New York and Maurice Choux in Marseille that new classifications and the first clinical series of children operated on for brainstem tumors were published [35–40]. This new era in brainstem surgery was facilitated by the systematic use of microsurgery, the advent of MRI—which allowed much more detailed information on the tumor characteristics before surgery—and a remarkably improved neurointensive care.

In the early 1990s, there was also an extended attempt, by neurosurgeons, to identify anatomical landmarks on the floor of the fourth ventricle to define safe surgical corridors to approach intrinsic brainstem tumors [41–44]. However, anatomical landmarks are extremely valuable when anatomy is preserved but can be unreliable if anatomy is distorted by the mass effect induced by the tumor itself.

Therefore, some neurophysiological techniques were needed to provide neurosurgeons with functional rather than merely anatomical landmarks. Some preliminary studies were published by Strauss et al. who used neurophysiological mapping to identify cranial nerve nuclei on the floor of the fourth ventricle [45, 46]. But, again, the contribution of pediatric neurosurgeons to the development of these techniques was substantial.

In the early 1990s, a young neurophysiologist from Croatia, Vedran Deletis, joined the pediatric neurosurgery team at the New York University (NYU) led by Fred Epstein, to develop intraoperative neurophysiological techniques that could improve the safety of neurosurgical procedure in children, particularly with regard to brainstem and intramedullary tumor surgery. Nobuhito Morota, a young neurosurgeon from Japan, was one of the first Deletis’ fellows at NYU. In 1995, for the first time, Morota systematically compared anatomical findings in a series of 14 patients with brainstem tumors, with the intraoperative validation provided by direct brainstem mapping [47]. He clearly showed that structures such as the nuclei of the facial nerve or those of the lower cranial nerves could be identified much more reliably through the use of neurophysiological mapping techniques, remarkably improving the localization of brainstem functional anatomical landmarks.

In a subsequent study, the following year, Morota et al. investigated the patterns of displacement of brainstem nuclei according to the tumor location [48]. Although this study was on a small series of 18 patients and was never replicated by others, still, it provided a valuable reference. To sum up, the conclusions were that upper pontine glioma tends to displace the facial nuclei downward and laterally, while lower pontine glioma does the opposite (upward and laterally). Medullary tumors, instead, tend to displace the lower cranial nerve nuclei more ventrally.

The surgical implications of those publications were relevant for avoiding facial palsy and, even more, injury to the glossopharyngeal/vagus and hypoglossal nuclei as this would impair the coughing and swallowing reflexes, ultimately exposing patients to life-threatening conditions such as aspiration pneumonia and respiratory insufficiency. What we learned from those studies was that as soon as direct neurophysiological mapping of the floor of the fourth ventricle, with low stimulation intensities, provides a positive response from any of the muscles innervated by these cranial motor nerves, surgery should stop. At this point, the surgeon should consider the intensity of stimulation and the type of stimulator in use. Typically, intensity up to 2 mA is used for direct brainstem mapping, avoiding stronger stimulations, particularly at the level of the medulla for the risk of inducing bradycardia and even cardiac arrest. The lower the threshold intensity for stimulation, the closer the nuclei (or the intramedullary roots) of these nerves, with 0.1–0.2 mA to be considered low thresholds. A concentric bipolar stimulator is preferable to a monopolar stimulation as it reduces the current spreading and therefore retains a higher localizing value.

In children, intrinsic or dorsally exophytic medullary gliomas are usually of low grade. Therefore, whenever towards the end of the resection a positive mapping of the lower cranial nerves occurs, one should carefully balance the oncological and functional risks, very similar to the concept of onco-functional balance in adult glioma surgery. To pursue a complete resection may expose to the risk of inducing permanent injury with the need for a, at least transient, tracheostomy and/or gastrostomy. This may be unjustified if the tumor is in the range of low-grade gliomas where a small remnant may remain indolent for many years or even disappear spontaneously. A different perspective applies to fourth ventricle tumors infiltrating the floor, such as medulloblastomas and, above all, ependymomas. Given the importance of total resection in posterior fossa ependymomas, here, the decision of whether or not to abandon surgery in the light of positive neurophysiological mapping may be more intriguing and controversial.

When it comes to intraoperative neurophysiological mapping strategies to remove brainstem tumors, pediatric neurosurgeons are certainly indebted to the studies by Morota et al., which remain seminal papers in this field. Nowadays, these techniques are successfully applied also to very young children [49].

One of the problems of mapping techniques, however, is that these allow the identification of safe corridors to approach intrinsic brainstem lesions but do not provide a continuous functional assessment of the integrity of the long pathways passing through the brainstem. For this, monitoring techniques are needed. In the early days, only SSEPs and brainstem auditory responses (BAERs) were available but these could not assess more than 20% of the brainstem and therefore motor or cranial nerve deficits are possible even with the preservation of SSEPs and BAERs [50]. At the time of our review paper on “why, when and how” to monitor pediatric neurosurgery, in 2002, the use of MEPs, and particularly corticobulbar MEPs, in brainstem surgery was still anecdotal. We wrote: “The possibility of monitoring corticobulbar pathways by recording MEPs from the facial, laryngeal and tongue muscles after transcranial stimulation is currently under investigation and may prove to be a useful monitoring tool in the near future” [19]. Since then, several studies have addressed the value of corticobulbar MEPs, which is nowadays a standard technique in brainstem and cerebello-pontine angle surgery, although most of the studies refer to the adult rather than pediatric population [51–54].

Finally, it should be stressed that robust techniques to monitor the afferent (sensory) pathways for the lower cranial nerve-mediated reflexes such as swallowing and coughing have been lacking for many years. This was problematic because an iatrogenic injury to the afferent arch of these reflexes would not be recognized by current monitoring and mapping techniques resulting in false negative results, namely patients with post-operative impairment of coughing and swallowing despite preserved intraoperative corticobulbar MEPs for the lower cranial nerves [55].

ION of reflex circuits within the brainstem is nowadays a new strategy to indirectly assess the functional integrity of these pathways. One example is the so-called laryngeal abductor reflex (LAR), which is mediated at the level of the lower brainstem [56]. The LAR is elicited by applying electrical stimuli to the laryngeal mucosa through electrodes placed on the endotracheal tube. The afferent arc carries information from the sensory receptors of the supraglottic mucosa to the brainstem via the internal branch of the superior laryngeal nerve. The efferent arch of the LAR provides motor innervation to the adductor laryngeal muscles via the recurrent laryngeal nerve. Therefore, preservation of the LAR likely reflects preservation of the lower brainstem. Initially used in thyroid surgery to preserve the superior laryngeal nerve, there are now some preliminary results on its application during surgery in the brainstem and the cerebello-pontine angle, including two pediatric cases [57].

## Spinal cord surgery (Fig. 3)

**Fig. 3:**
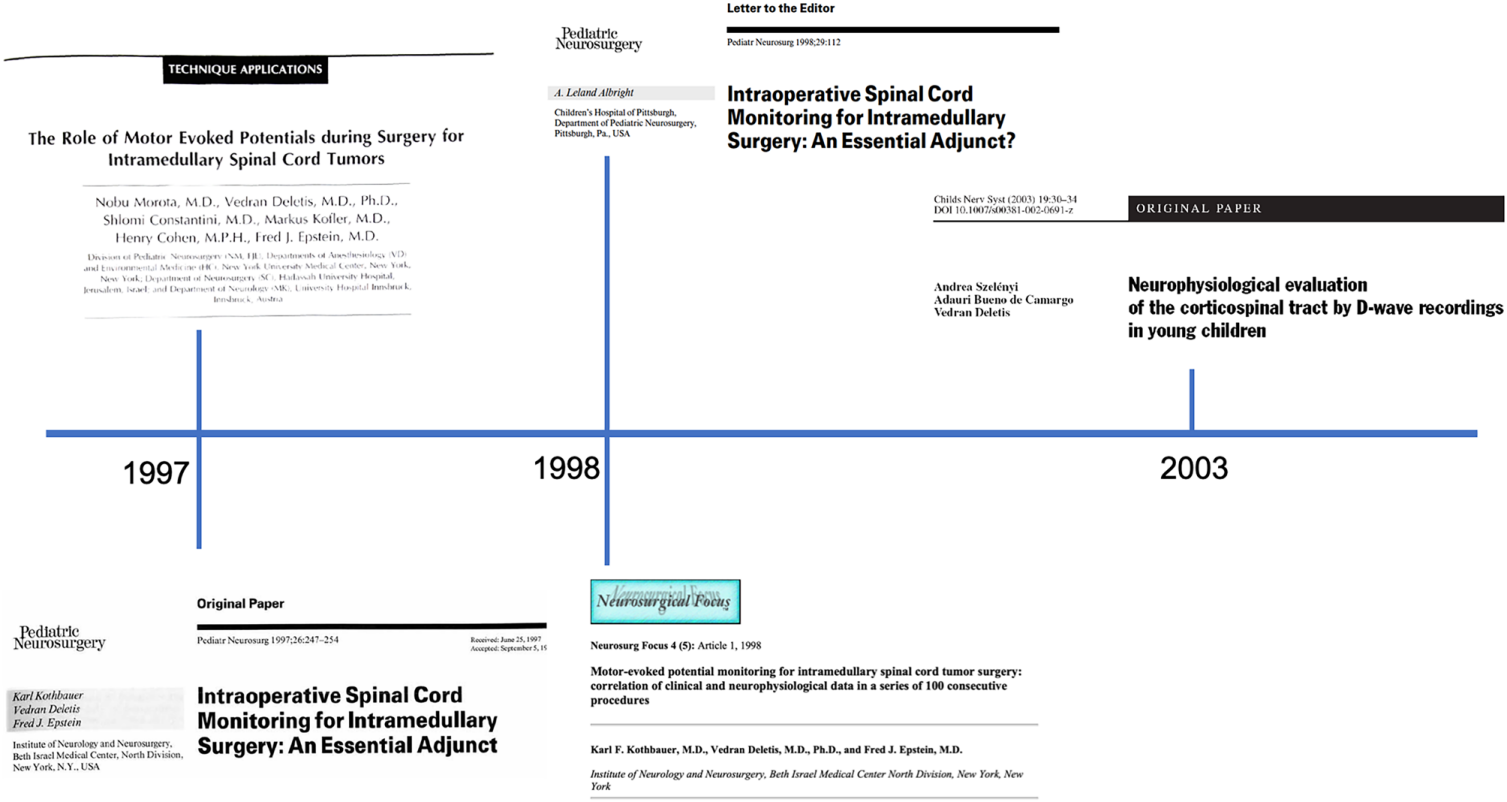
Timeline of ION development in spinal cord tumor surgery

Similarly to brainstem surgery, intramedullary spinal cord tumor (ISCT) surgery has always been considered very challenging due to the high functional relevance of this tiny neural structure. C. Helsberg in his 1925 book “Tumors of the Spinal Cord” wrote that “…no matter how markedly the tumor will seem to bulge, the surgeon must not attempt to remove the growth, for he will be sure to cause grave injury to the cord. He must leave it to nature to extrude the tumor.” In the following decades, the attempt to remove intrinsic spinal cord tumors was abandoned in favor of a more conservative approach limited to biopsy and radiotherapy. It was only in the early and mid-1980s that neurosurgeons re-considered surgery as the first option for some of these lesions. Within a few years, J. Brotchi in Brussels, G. Fischer in Lyon, P. McCormick and F. Epstein in New York, among others, published surgical series of ISCTs with Epstein [58–61], in particular, publishing the first pediatric series [62, 63].

As the surgical treatment of ISCTs was challenging and burdened by serious complications, the partnership between pediatric neurosurgeons and neurophysiologists proved to be, once again, very successful. Initially at NYU and, subsequently, at the Beth Israel Institute for Neurology and Neurosurgery in New York, two neurosurgery fellows, N. Morota and K. Kothbauer, decided to invest their training in both pediatric neurosurgery and intraoperative neurophysiology, under the mentoring of F. Epstein and V. Deletis. Between 1997 and 1999, they published two papers which represented the cornerstones for the subsequent development of ION in ISCT surgery. Morota, in 1997, introduced for the first time the intraoperative recording of the so-called D wave [64].

The D-wave represents the specific activation of the fast-conducting fibers of the corticospinal tract (CST). These amounts for no more than 2% of all CST fibers but are those of the largest diameter and fastest conduction velocity, thanks to their high degree of myelinization. The preservation of the D-wave amplitude, considering a cutoff value of 50%, has proved to be the strongest predictor of good long-term motor outcome. In our own experience (unpublished data), the D-wave was preserved at the end of surgery in 19 out of 20 children (95%) operated for ISCTs. This warranted a McCormick grade of I or II (namely, able to walk without assistance) in 93% of them at a mean follow-up of 12 months after surgery. Despite the fact that only a few studies have specifically addressed the reliability of D-wave monitoring in predicting motor outcome [65], all these studies have consistently confirmed the highly predictive value of this neurophysiological parameter [64, 66–69].

One year after Morota’s study, Kothbauer published in *Neurosurgical Focus* the first series of 100 ISCTs all operated with ION [67]. This study has remained for many years the largest series of ISCT patients, adults and children, consistently operated with ION, showing that a combination of D-wave and muscle MEP monitoring warranted a remarkable sensitivity (100%) and specificity (91%).

In 1997, another paper was published by Kothbauer in *Pediatric Neurosurgery* [70]. The rather provocative title “Intraoperative Spinal Cord Monitoring for Intramedullary Surgery: An Essential Adjunct” solicited an Editorial by L. Albright, who sagaciously added just a question mark at the end to dispute the evidence for the value of IONM [71]. He wrote: “Lawyers are not known for their close reading of the medical literature and some will no doubt latch onto this article as indicating that monitoring SEPs and MEPs are the standard of care for all who operate on children with intramedullary tumors. Yet SEPs are of no value in predicting postoperative motor function and although MEPs may well be of value, we have not yet seen confirmatory data that they are.”

At that time skepticism about the reliability of ION was still quite diffuse, but Dr. Albright pointed out something which became more and more relevant in the following two decades, namely the debate on the medico-legal implications of neuromonitoring. In 2017, more than 20 years later, A. Jea in an Editorial in *Neurosurgical Focus* entitled: “Intraoperative Neuromonitoring: gold standard or fool’s gold” critically reviewed a paper by Zuccaro et al. [72] addressing the value of ION during surgery for spine deformity in children [73]. In his editorial, he mentioned that “the authors are too aggressive in suggesting that IONM is “standard of care”; it implies that spine surgeons who do not use IONM are committing malpractice.” Almost 30 years have gone by since that respectful debate on the value of ION, which took place in a pediatric neurosurgical journal and, to some extent, that discussion is not completely over. Yet, despite the persistence of believers and disbelievers towards the value of ION and the evidence for it, it should be acknowledged that nowadays, there is a large consensus on the fact that ION is indeed essential when it comes to ISCT surgery [74]. While the evidence for the use of ION in most extramedullary neurosurgical procedures remains debated and the indication for ION is optional, in 2023, it would be both ethically and medico-legally arguable to operate on an ISCT without ION.

In the very same paper on ION in children with ISCTs, Kothbauer et al. observed that children below the age of 9–10 years often retained SSEPs regardless of the magnitude or width of the myelotomy, speculating that in young children, intramedullary spinal cord tumors may develop already during gestation, therefore displacing dorsal columns more laterally. This assumption was never confirmed but it may be worthy to investigate it nowadays using the more recent techniques for direct mapping of the dorsal columns [70].

One of the intriguing aspects of D-wave monitoring remains its poor monitorability in younger children. The role of age was investigated in 2003 by Szelenyi et al. who reviewed D-wave monitoring in 19 children operated for ISCTs below the age of 3 years [75]. A D-wave was recorded in only 7 children aged between 21 and 36 months but not in 12 children aged between 8 and 31 months. The youngest patient with a recordable D-wave was 21 months old. The current interpretation for the poor monitorability of the D-wave in younger children is that the immaturity of their corticospinal tracts accounts for a desynchronization of the descending volleys elicited by the transcranial stimulation to the point that an epidural catheter on the spinal cord cannot record a synchronized, measurable, signal. Yet, the temporal and spatial summation of these volleys at the level of the α-motoneuron still warrants a muscle contraction. Also, these D-waves were recorded caudally to the tumor site, and it was therefore difficult to differentiate between the effect of the CST immaturity and that of the tumor itself.

Recently, Antkowiak et al. published a series of 23 children with ISCT operated with ION. D-wave was monitorable in 60.9% of the cases and retained a specificity of 92.3% and a sensitivity of 100%, accurately predicting post-operative motor deficits [76]. They also concluded that ION did not limit the extent of resection (GTR in 29.4% of patients without ION alerts and in 33.3% of those with ION alerts), confirming our own experience in a larger series of adult patients with intramedullary ependymomas [69].

## Tethered cord surgery (Fig. 4)

**Fig. 4:**
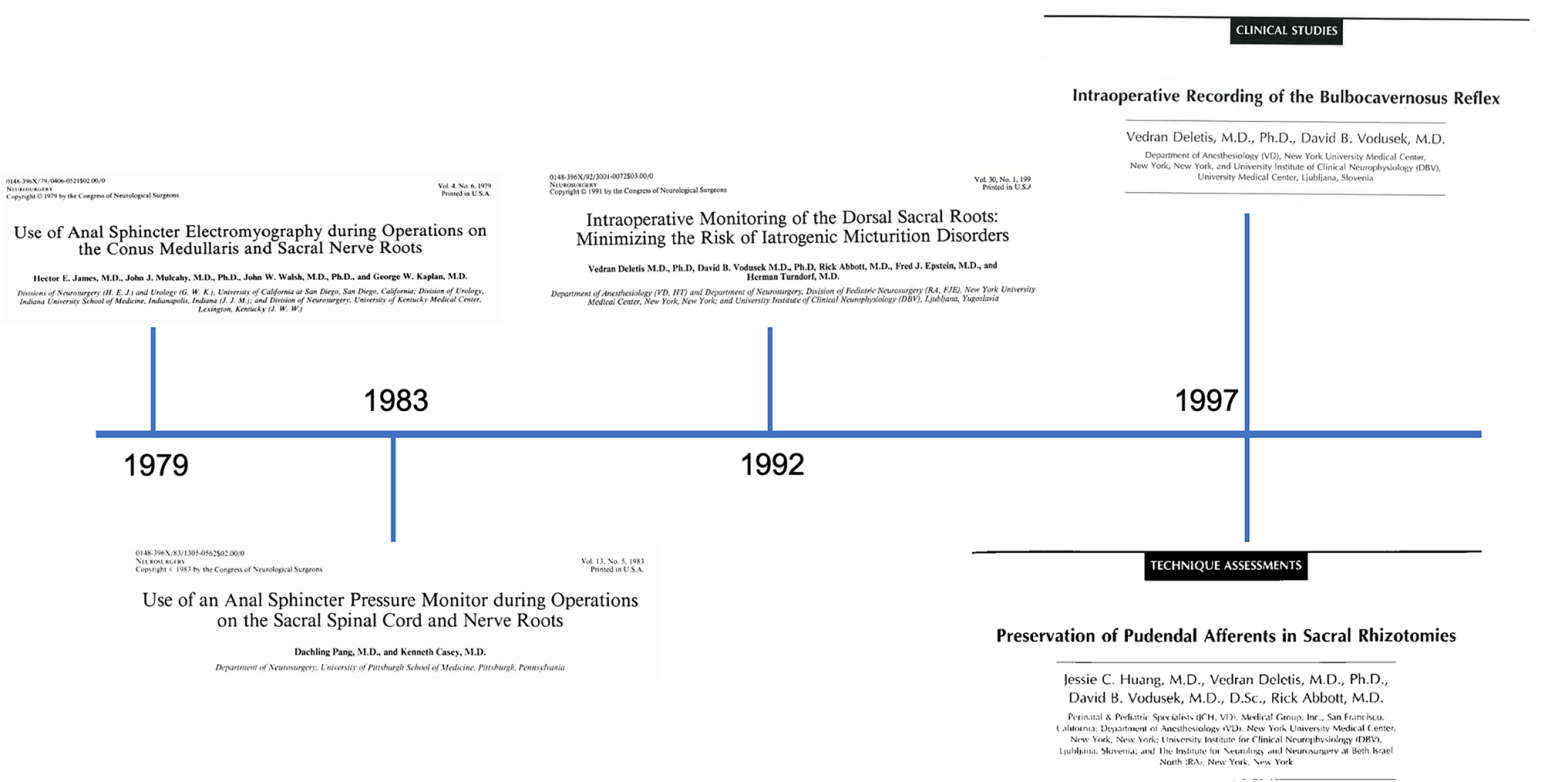
Timeline of ION development in tethered cord surgery

Surgery in the conus-cauda equina region exposes to the risk of sensorimotor deficit to the lower extremities and sphincterial deficits. To minimize surgical morbidity, ION techniques have been in use for many decades during surgery for occult spinal dysraphisms. Traditionally, both spontaneous EMG as a monitoring technique and triggered EMG as a mapping technique have been used. Triggered EMG is based on the electrical stimulation of a motor nerve root and the recording of a compound muscle action potential from the innervated muscle. In the early and mid-1990s, these techniques were in use both in Europe [77] and North America [78]. Free-running EMG was the standard, and MEPs were not yet available in most centers. Later on, with the advent of the train-of-five technique, transcranial MEPs recorded from lower extremity muscles, including the anal sphincter, became of routine use also in conus-cauda surgery, although free-running EMG still retains its value today and can be indicative of lower motor neuron damage [79].

Apart from monitoring MEPs and SEPs from the lower extremities, ION techniques to monitor sphincter function are of paramount importance during conus-cauda surgery. In the late 1970s, James et al. proposed the use of EMG recordings from needle or plug electrodes inserted in the anal sphincter to identify and preserve sacral pudendal nerve roots [80]. They assumed that the activity of the anal sphincter could be extrapolated to that of the external urethral sphincter, both being striated muscles innervated by the pudendal nerves. Four years later, as an alternative to EMG, Pang and Casey proposed the use of an anal sphincter pressure monitor as a noninvasive method of monitoring the external anal sphincter “squeeze pressure” by means of a polyethylene anal balloon connected to a pressure transducer [81]. They observed that unilateral stimulation of the S-2, S-3, and S-4 roots generated tall pressure spikes between 40 and 75 torr in peak amplitudes, unlikely from the L5 and S1 roots, and found it a reliable technique to map rootlets innervating the anal sphincter. They preferred this technique “because of its simplicity, the inexpensive equipment, and its noise-free display that is virtually unaffected by other electronic systems in the operating room.”

Both in tethered cord surgery and in selective dorsal rhizotomy (SDR) for spasticity, the identification of pudendal afferents from the dorsal penile and clitoral nerves is important to avoid disorders of micturition. A technique to selectively identify these pudendal nerves was developed by Deletis et al. in 1992 [82]. They studied 31 children and one adult who underwent surgery in the cauda equina and recorded pudendal afferents directly from the exposed S1-S3 sacral roots. Interestingly, the distribution of pudendal afferents varied across different patients but was concentrated on S2 and S3, with S1 never being the main carrier. Also, not in every child the S2 rootlet was carrying substantial afferent activity, which introduced the possibility to extend SDR to S2 in selected cases, without the risk of postoperative incontinence. This preliminary experience was corroborated by a much larger study, from the same authors, in 1997 when they reported no long-term bowel or bladder disturbances in 105 out of 114 undergoing SDR, where pudendal afferent mapping was successfully used [83].

Similarly to other fields in ION, the different implications of mapping and monitoring techniques are relevant also during surgery in the conus-cauda region. Mapping techniques allow the functional identification of anatomically ambiguous neural structures. This can be invaluable whenever anatomy does not suffice to provide functional information, for example discriminating between functional and non-functional rootlets encased in a lumbosacral lipoma or between a real rootlet and a non-functional fibrous band. Maybe the most dramatic example of the value of ION as a mapping technique in tethered cord surgery is that of the retained medullary cord, where only neurophysiological mapping allows to discriminate between the functional conus and sacral roots versus the retained medullary cord, which has lost its function [84, 85].

Yet, mapping techniques do not provide any information on the functional integrity of neural pathways between two consecutive mappings, and monitoring techniques should be used.

In this perspective, while mapping techniques for afferent and efferent pudendal nerves were available from the late 1970s and further developed in the 1990s, a monitoring technique for these nerves was not available until the mid-1990s.

In 1997, Deletis and Vodusek demonstrated for the first time the feasibility of recording the bulbocavernosus reflex intraoperatively, under general anesthesia [86]. The technique was further refined by Rodi and Vodusek in 2001 and rapidly became very popular within the ION community [87]. This reflex allows us to monitor the integrity of the S2 to S4 motor and sensory roots, as well as the S2 to S4 spinal cord segment. The BCR is extremely sensitive to anesthesia and becomes unstable due to manipulation at various points along its pathway, sometimes without a clear clinical correlate [88, 89]. Therefore, it is sometimes difficult to correlate intraoperative BCR results with the post-operative functional outcome. While preservation of the BCR is predictive of good post-operative urinary function, the complete loss is usually indicative of at least transient urinary retention (rarely incontinence), which mostly, but not necessarily, resolves with time. Yet, amplitude warning criteria for the BCR are still debated. Recently, Morota proposed a reduction of > 75% from baseline amplitude as a criterion to alert the surgeon to modify the procedure for preserving urinary function [90]. Overall, interpretation of BCR monitoring remains challenging because bladder contraction is controlled by parasympathetic fibers and not by the pudendal nerve. These fibers control urethral detrusor contraction and relaxation of the internal urethral sphincter, thus permitting the bladder to void. Therefore, separate monitoring of the detrusor muscle would be more reliable to detect impairment of bladder control. The detrusor muscle integrity can be evaluated by changes in urinary bladder pressure measured with a manometer connected to a Foley catheter [91]. With these limitations in mind, BCR monitoring remains a valuable technique and Morota reported no urological complications in 118 patients where BCR amplitude at the end of surgery diminished less than 50% of baseline amplitude [90].

In tethered cord surgery, one of the most debated topics over the past two decades remains the surgical indications for asymptomatic lumbosacral lipomas. Traditionally, much of the debate has focused on the comparisons between the Necker study on the natural history of conus lipomas [92] treated conservatively versus Pang’s series where gross total resection of the lipomas was achieved [93]. What surprises us is that very rarely the role played by ION was taken into consideration when discussing the results of these studies. In fact, ION was systematically used in Pang’s series and never used in the French study, which could be per se a variable explaining the attempt of near-total resection proposed by Pang and the more conservative attitude in the lack of any neurophysiological guidance.

## Future perspectives and conclusions

If we look 20 years back and consider the review we published in 2002 on the “Why, when and how” to perform ION in *Pediatric Neurosurgery* [19], we can conclude that the vast majority of the ION techniques used at the beginning of this century are still valid nowadays. The main difference is that today, most of these techniques have been consolidated on a much larger series of patients and are now of common use in most pediatric neurosurgical centers and not limited to a few institutions, as it was at the end of the 1990s and early 2000s.

When dealing with the pediatric population, one should always consider that the maturation of the nervous system is still in progress, and, particularly for motor pathways, there is a continuum of maturation throughout adolescence. This requires special adjustments of the ION techniques used in adults [94].

Looking at the future, two main fields of research may open new perspectives in pediatric neurosurgery. One is the recording of cortico-cortical evoked potentials (CCEPs). These have been increasingly investigated in the past few years, mainly from a methodological and feasibility standpoint and in adult patients. The principle of CCEPs is to monitor direct connectivity between two separate areas of the cortex. The best example is the monitoring of the arcuate fascicle by stimulating from the frontal lobe and recording from the temporal cortex and vice versa. This was originally investigated by Matsumoto et al. in 2004 in the awake setting, studying eight patients who underwent chronic subdural electrode placement for the presurgical evaluation of medically intractable partial epilepsy [95]. More recently, a similar approach has been proposed intraoperatively in the asleep setting, by different groups [96–98]. As much as these results are still very preliminary, the possibility to assess intraoperatively the functional integrity of the arcuate fascicles and, therefore, monitor and possibly preserve this important pathway in language production has obvious implications in pediatric neurosurgery where awake craniotomy is rarely feasible.

The other fascinating field is the intraoperative neurophysiology of the cerebellum, which has been ignored for many years by both neurophysiologists and neurosurgeons [99]. A few, still very preliminary and anecdotal reports have been published with regard to the sensorimotor function of the cerebellum [100, 101]. Also, a method to possibly monitor the so-called dentate-thalamic-cortical pathway involved in the pathophysiology of cerebellar mutism is currently under investigation [102].

These techniques need further methodological fine-tuning and reproducibility and, above all, will need validation in clinical studies comparing the intraoperative neurophysiological data with the clinical outcome. Nevertheless, these still represent novel, fascinating techniques to explore connectivity within the central nervous system and have potential applications in pediatric neurosurgery.

ION has evolved dramatically over the past 50 years. The golden age of ION took place during the 1990s when we witnessed the highest development of novel techniques to map and monitor the central and peripheral nervous system. Pediatric neurosurgeons have played a major role in this endeavor contributing to the development of many of these techniques. The field of ION is, yet, still in evolution, and new techniques are currently being investigated, hopefully offering new opportunities to increase the safety of pediatric neurosurgical procedures.
